# Protocol for a systematic review of the diagnostic and prognostic utility of tests currently available for the detection of aspirin resistance in patients with established cardiovascular or cerebrovascular disease

**DOI:** 10.1186/2046-4053-2-16

**Published:** 2013-02-26

**Authors:** Smriti Raichand, David Moore, Richard D Riley, Marie Lordkipanidzé, Janine Dretzke, Jennifer O’Donnell, Sue Jowett, Sue Bayliss, David A Fitzmaurice

**Affiliations:** 1 Public Health, Epidemiology and Biostatistics, School of Health and Population Sciences, University of Birmingham, Edgbaston, Birmingham, B15 2TT, UK; 2 School of Clinical and Experimental Medicine, College of Medical and Dental Sciences, Institute of Biomedical Research, University of Birmingham, Edgbaston, Birmingham, B15 2TT, UK; 3 Health Economics, University of Birmingham, Edgbaston, Birmingham, B15 2TT, UK; 4 Primary Care Clinical Sciences, University of Birmingham, Edgbaston, Birmingham, B15 2TT, UK

**Keywords:** Aspirin resistance, Cardiovascular disease, Cerebrovascular disease, Diabetes, Diagnosis, Meta-analysis, Platelet function test, Prediction, Prognosis, Test accuracy

## Abstract

**Background:**

The benefits of aspirin as an anti-platelet agent are well established; however, there has been much debate about the lack of uniformity in the efficacy of aspirin to inhibit platelet function. In some patients, aspirin fails to inhibit platelets even where compliance has been verified, a phenomenon which has been termed “aspirin resistance”. These patients may in turn be at a higher risk of future vascular events. The proportion of “resistant” patients identified depends on the type of platelet function test. Therefore, the aim of this systematic review is to determine which, if any, platelet function test has utility in terms of identifying patients with a high risk of vascular events. The review has been registered with PROSPERO (CRD42012002151).

**Methods:**

Relevant studies will be sought from bibliographic databases. Trials registers will be searched for ongoing studies. Reference lists will be checked and subject experts contacted. There will be no date or language restrictions. Standard reviewing methodology to minimise bias will be employed. Any prospective studies in patients on aspirin therapy and assessing platelet function in relation to relevant clinical outcomes will be included, as will studies reporting prognostic models. Risk of bias assessment will be based on the Quality Assessment of Diagnostic Accuracy Studies guidelines, and suitable criteria for assessing quality of prognostic studies. Data on test accuracy measures, relative risks, odds or hazard ratios will be extracted and meta-analysed, where possible, using a random-effects model to account for between-study heterogeneity. Where appropriate, the causes of heterogeneity will be explored through meta-regression and sub-group or sensitivity analyses. If platelet function testing is demonstrated to have diagnostic/predictive utility in a specific population, the potential for a cost-effectiveness analysis will be considered and, if possible, an economic model constructed. This will be supported by a systematic review of existing economic evaluation studies.

**Discussion:**

The results of the review could indicate if platelet function test(s) could lead to a reliable prediction of the risk of clinically important events in a defined population, and thus support investigations into adjustments to therapy in order to compensate for a predicted poor response to standard aspirin.

## Background

The clinical efficacy of aspirin as an antiplatelet agent to prevent occlusive arterial events in patients with atherothrombotic disease is well established; daily administration of aspirin has been shown to reduce the risk of stroke, myocardial infarction (MI) and death by approximately 25% [1]. Despite these proven benefits, many patients continue to experience thrombotic events. Several factors may influence the response of platelets to antiplatelet therapy [2], including treatment adherence [3]. However, in some patients even in the context of verified compliance, aspirin fails to inhibit platelets as determined by *ex vivo* laboratory tests, a phenomenon termed “resistance” to antiplatelet therapy [4].

Many platelet function tests have been used to assess inhibition of platelet function induced by aspirin and their methodologies are diverse [5,6]. While some assays study global haemostasis, most platelet function assays target a specific phase of platelet function, from platelet adhesion to platelet activation, secretion and aggregation [7,8]. Methodological differences make it difficult to compare the test results. For example, some of these assays are carried out in whole blood (including whole blood aggregometry, platelet counting, PFA-100, VerifyNow, Impact-R and flow cytometry), while others require sample preparation (such as plasma or serum thromboxane B_2_ measurement), and others can be performed on urine (levels of the thromboxane B_2_ metabolite 11-dehydro-thromboxane B_2_) [9]. Thresholds to determine whether platelet function is within a normal range may vary between studies of the same test depending on how the threshold was established (*e.g.*, thresholds commonly reported in the literature, prescribed by the manufacturer or derived from comparisons with healthy control populations).

Most importantly, some assays are specific to the action of aspirin, by using an agonist that targets the platelet pathway inhibited by aspirin (the cyclooxygenase pathway) or by measuring metabolites of thromboxane A_2_ (which is the end-product of the pathway blocked by aspirin), while other platelet function assays assess pathways not directly affected by aspirin but measure global platelet reactivity [5,10,11]. The effect of aspirin is, of course, more obvious in aspirin-specific assays, but the global platelet reactivity assays could be more clinically relevant as they capture a more global portrait of platelet function [12]. However, for such global assays to be able to determine resistance to aspirin therapy would require aspirin to be the only platelet function modifying agent used by the patient.

There is no official guideline recommending one assay above another, and platelet function testing is not recommended for routine clinical testing in patients requiring aspirin therapy [13]. As a result, many of the available platelet function assays have been used in a research capacity, and part of the uncertainty surrounding the definition and clinical relevance of aspirin resistance is due to the non-interchangeable nature of these assays [14]. As a consequence, there is a need to address basic questions on the prognostic and diagnostic utility and cost-effectiveness of platelet function testing in the context of aspirin therapy before testing can be recommended in clinical practice.

### Existing systematic reviews

Three relevant recent systematic reviews were identified, all of which included a meta-analysis [15-17]. The review by Reny *et al.*[17] looked at the PFA-100 test only to predict cardiovascular events in patients with symptomatic atherosclerosis, and included 8 prospective studies. A statistically significant result was reported for an increased risk of ischemic events in patients defined as ‘aspirin resistant’ by the test. The other reviews included a range of platelet function tests and identified 17 studies [15] and 20 studies [16] respectively. Both reviews reported a statistically significant increased risk of cardiovascular events in patients with ‘aspirin resistance’ as defined by the laboratory test(s).

There were some methodological limitations associated with the latter two reviews, including a lack of detail on quality assessment of included studies, the thresholds (cut-off points) used to define ‘aspirin resistance’ for each test, and patient compliance. The diagnostic utility of the tests (*e.g.,* in terms of sensitivity and specificity) was also not considered. Further, heterogeneity between included studies due to differences in platelet function assays, study follow-up periods and aspirin dose, needs to be accounted for and, where possible, investigated in more detail. Since the completion of these reviews, several further studies have been published, particularly due to the emergence of dual/triple antiplatelet therapy regimens (where aspirin is one agent) for treating some at-risk populations with cardiovascular disease, such as those with acute coronary syndrome or undergoing percutaneous coronary intervention.

The aim of the current project is to perform a new systematic review that, in comparison to existing reviews, additionally incorporates any missed studies and/or studies published more recently; implements a more robust and complete meta-analysis of the evidence, including an improved approach to quantifying and examining clinical and statistical heterogeneity; and summarises the evidence in relation to both prognostic association (*e.g.*, odds ratios, hazard ratios) and diagnostic/predictive test accuracy (*e.g.*, sensitivity, specificity) for individual patients.

### Research aims

This systematic review will look at studies which relate platelet function testing to the risk of adverse clinical outcome(s) in patients on aspirin therapy with established cardiovascular or cerebrovascular disease, or diabetes. More specifically, the review will aim to determine the diagnostic/predictive utility and the prognostic utility of different platelet function tests:

i) Diagnostic/predictive utility:

to establish whether any of the available platelet function tests to determine “aspirin resistance” has sufficiently high diagnostic/predictive utility (*e.g.*, sensitivity, specificity and positive and negative predictive values close to 1) in order to determine, for individual patients, if treatment modification should be considered based on the test result;

ii) Prognostic utility:

to establish whether any of the available platelet function tests has prognostic ability, *i.e.*, whether it is able to distinguish between groups of patients with different average outcome risks even if it does not accurately predict individual outcome risk.

Further, the potential for a cost-effectiveness analysis will be considered. An economic model will be constructed and the potential for populating the model with data based on the results of the systematic review explored.

## Methods

This protocol is registered with PROSPERO (2012:CRD42012002151).

### Selection criteria

i) Diagnostic/predictive utility and prognostic utility studies

#### Types of study

Any prospective primary studies, or systematic reviews of such studies, assessing platelet function test(s) in relation to clinical outcomes.

#### Types of participants

Patients aged ≥18 years on aspirin (as monotherapy or in combination with other antiplatelet agents), with established cardiovascular or cerebrovascular disease, or diabetes. Studies with mixed populations will be included as long as data for relevant patients is extractable. Studies with patients on aspirin for peripheral vascular disease will be noted.

#### Setting

Studies in any setting will be included.

#### Technology

Either aspirin specific platelet function test or global platelet function test where patients are receiving aspirin as the only antiplatelet therapy; *or* aspirin specific platelet function test where patients are on dual/triple antiplatelet therapy (with aspirin as one of the agents).

#### Outcomes

Clinical outcomes, such as: vascular events (non-fatal and fatal ischemic stroke, transient ischemic attack, systemic embolism (pulmonary embolism, peripheral arterial embolism), MI, stent thrombosis); mortality due to vascular events.

Other outcomes will include:

ii) Prognostic model studies

There may be studies that report prognostic models, in which a platelet function test is one of multiple prognostic factors predicting clinical outcomes in a population of interest. In order to examine the contribution of the test to the overall performance of the prognostic model, and to establish whether predictive accuracy of clinical outcomes is improved by combining test results with other prognostic factors, the studies will need to meet the following inclusion criteria:

i. Was a statistical model outlined to predict a relevant clinical outcome?

ii. Did the model include a factor for platelet function test or aspirin resistance?

iii. Was the model developed for use in patients ≥18 years old and on aspirin (alone or in combination with another therapy) for established cardiovascular, cerebrovascular disease or diabetes?

All-cause mortality; bleeding (categorised according to the International Society of Haemostasis and Thrombosis as major bleeding events, clinically relevant non-major bleeding events or minor bleeding events); major adverse cardiovascular events (composite of all-cause mortality, non-fatal MI and stroke); revascularisation procedures (percutaneous coronary intervention, coronary arterial bypass graft, embolectomy).

### Search strategy

Bibliographic databases (Cochrane Library, MEDLINE, EMBASE) will be searched from inception to 2012. Searches will use combinations of index and text words that encompass the technology and the patient group supplemented by aspirin resistance terms (see sample MEDLINE search in Appendix 1). The ZETOC database (British Library) and the Science Citation Index (Web of Science) will be searched for conference proceedings. Publicly available trials registers, such as ClinicalTrials.gov, UK Clinical Research Network Study Portfolio Database (UKCRN), WHO International Clinical Trials Registry Platform and the *meta*Register of Controlled Trials (*m*RCT) will be searched for ongoing studies. Reference lists of all included papers will be checked and subject experts will be contacted. No restrictions on publication language and date of publication will be applied.

In addition, abstracts from the following national and international proceedings will be consulted from 2009 onwards in order to capture studies that are not yet fully published:

•Platelet conferences (Platelets International Symposium);

•Cardiology conferences (British Cardiovascular Society, American College of Cardiology (ACC), European Society of Cardiology (ESC), American Heart Association (AHA), American College of Chest Physicians (ACCP));

•Stroke conferences (International Stroke Conference, American Stroke Association);

•Haematology conferences (British Society for Haematology, International Society on Thrombosis and Haemostasis, International Society for Laboratory Haematology).

### Study selection

This will be a two-step process. Titles (and abstracts where available) will initially be screened by two reviewers, using pre-specified screening criteria. These are broadly based on whether the studies 1) are about platelet function testing, 2) report clinical outcomes, and 3) are in patients on aspirin therapy. Full texts of any potentially relevant articles will then be obtained and two reviewers will independently apply the full inclusion criteria. Any discrepancies between reviewers will be resolved by discussion or by referral to a third reviewer. Appropriate portions of non-English language articles will be translated where necessary. The study selection process will be illustrated using a PRISMA flow diagram [18]. Reference management software (Reference Manager v11 Thomson ResearchSoft) will be used to record reviewer decisions, including reasons for exclusion.

### Data extraction

Data extraction will be conducted independently by two reviewers using a standardised, piloted data extraction form. Disagreements will be resolved through discussion or referral to a third reviewer.

Data extraction will include the following variables:

•Study characteristics (*e.g*., country, year, sample size, number of centres);

•Study design (*e.g*., RCT, case-series, risk of bias);

•Patient characteristics (*e.g*., level of cardio- or cerebrovascular risk in those designated aspirin resistant or sensitive);

•Antiplatelet regimens (*e.g*., dose, compliance with clinical guidelines, co-interventions, patient adherence to treatment);

•Platelet function test utilised (*e.g*., thresholds, other relevant laboratory parameters);

•Outcome measures and length of follow-up (all vascular events, including MI, acute coronary syndrome, ischemic stroke, transient ischemic attack or systemic embolism, cardiovascular death, all-cause mortality, adverse events, major and minor bleeding; revascularisation);

•Data required to complete a 2 × 2 table (see Table 1 below), otherwise relative risks, odds ratios or hazard ratios (with estimates of uncertainty [19]); where sufficient statistical information cannot be extracted, study authors will be asked to supply necessary information;

•Statistical methods employed and appropriateness of these.

**Table 1:** A 2x2 table to be extracted from each study for each binary outcome of interest

**Test result**	**Number of patients with an event**	**Number of patients with no events**
**Test positive (‘Aspirin resistant’)**	TP	FP
**Test negative (‘Aspirin sensitive’)**	FN	TN

TP = true positives; FP = false positives; FN = false negatives; TN = true negatives.

Data will also be extracted on any prognostic models, including results of models reporting prognostic risk with and without test results as a parameter.

### Assessment of risk of bias

Risk of bias will be assessed independently by two reviewers in accordance with Quality Assessment of Diagnostic Accuracy Studies (revised tool) (QUADAS-2) guidelines for diagnostic test studies [20]. As there is also a prognosis element, these guidelines will be supplemented with suitable criteria for checking the quality of prognostic studies as suggested by Hayden *et al.*[19]. Specific elements to be considered include:

•Study population (adequately described, details on sampling/recruitment);

•Study attrition (length of follow-up time, amount and timing of censoring, reasons for loss to follow-up);

•Test measurement (adequately described, timing of tests, missing tests results);

•Outcome measurement (adequately and unambiguously described, measured blind to the test result);

•Statistical analysis (whether continuous tests results were analysed on their continuous scale and, if not, whether a non-data-dependent explanation was given for the choice of thresholds to categorise the tests).

If any prognostic models are identified, the quality criteria described by Altman *et al*. [21] will be used in addition to those of Hayden *et al.*[19]. Specific elements to be considered include:

•Methods of model development (selection of candidate risk variables, relative weighting, handling of continuous variables);

•Internal and external model validations;

•Study design (prospective/retrospective);

•Sample size (considered *a priori*);

•Missing data (quantity, and how missing data was handled in the statistical analysis);

•Criteria for inclusion of prognostic factors into the model (adequately described, and whether well-known prognostic factors were included regardless of significance).

Any prognostic models identified will be summarised qualitatively (such as included variables; calculation of risk score; predictive accuracy; whether the model was validated internally and externally) and quantitatively by extracting performance statistics for calibration (such as Observed/Expected outcomes, O/E) and discrimination (such as sensitivity and specificity) of the model.

Similarly, if prognostic models report the incremental value of including tests, we will qualitatively summarise the added value reported in each study (such as net reclassification improvement, change in C statistic, etc.).

### Evidence synthesis

#### Diagnostic/predictive utility

Where possible, meta-analysis will be applied to synthesise the 2 × 2 tables from each study and produce summary estimates of test accuracy in terms of sensitivity, specificity, and positive/negative likelihood ratios for each platelet function test (and for each threshold specified if possible). The overall prevalence (absolute risk) of each outcome will also be summarised across studies, alongside the outcome prevalence in each test group separately. Analyses will be considered for a composite endpoint of all vascular events as the primary outcome, and then each type of vascular event individually as secondary outcomes.

A bivariate random effects meta-analysis model will be applied, where possible, using maximum likelihood estimation in STATA and Cochrane Revman (v5) software in order to obtain the aforementioned summary estimates of test accuracy measures. Between-study heterogeneity and correlation in sensitivity and specificity caused by different study threshold choices and other factors are accounted for in this method [22], and the exact binomial distribution of the data correctly modelled in each study [23]. Further, the summary receiver operating characteristic (ROC) curve, positive and negative predictive values for a range of outcome prevalences, and the summary diagnostic odds ratio will also be estimated. In order to give a range for the sensitivity and specificity of each test when applied to a specific clinical setting, 95% prediction intervals will also be calculated.

#### Prognostic utility

To summarise prognostic ability, where possible a random-effects meta-analysis model will be fitted to the extracted data using method of moments [24] to produce summary estimates of the relative risk and odds ratio (and their associated uncertainty) for each test. Where studies have reported results from a ‘time-to-event’ analysis to account for censored observations (such as a Cox regression), hazard ratios and their associated uncertainty will rather be extracted and then synthesised [25]. Where possible, each threshold will be summarised separately.

Both unadjusted and adjusted results will be extracted from each study, and a separate meta-analysis conducted for each where possible. Unadjusted results inform the prognostic utility of a test when used in isolation. Adjusted results rather reveal whether a test has prognostic utility over and above other prognostic factors; a true causal factor of poor outcome will retain strong prognostic value even after adjustment, and so this will also help to elicit the genuine clinical value of each test.

By default we choose a random effects meta-analysis model to account for likely between-study heterogeneity. Heterogeneity across studies will be measured statistically (using I^2^ and τ^2^) and examined qualitatively. Following each random-effects meta-analysis, a 95% prediction interval will also be calculated to estimate how the prognostic effect may vary from the average in different contexts and populations [26,27].

Where it is not appropriate to pool data, study results will simply be tabulated and described separately.

#### Test for funnel plot asymmetry

For each meta-analysis of prognostic utility containing 10 or more studies, the likelihood of publication bias will be investigated using contour-enhanced funnel plots [28] and appropriate statistical tests (such as the Peters Test [29]) for ‘small-study effects’; that is, the tendency for smaller studies to provide more positive findings [30].

#### Meta-regression and subgroup or sensitivity analyses

For each meta-analysis with sufficient numbers of studies (at least 10), sub-group analyses and/or meta-regression will be used to explore whether the following pre-specified variables explain any of the heterogeneity: population parameters, definitions of cardiovascular and/or cerebrovascular risk/ diabetes, aspirin dose, test method(s), adherence to treatment, type of outcome, length of follow up, study quality (risk of bias), and test threshold point employed. The sensitivity of the overall results to the inclusion/exclusion of particular studies will be examined. Where results for multiple thresholds exist for studies of diagnostic/predictive test accuracy, each threshold will first be meta-analysed separately, but then a sensitivity analysis will be undertaken according to the multivariate modelling framework of Riley *et al.* (personal communication), which analyses all thresholds together.

The key subgroup/sensitivity analysis of interest is for adherence to aspirin treatment. This will be achieved by either: i) combining only those studies that present results for patients who adhered to aspirin treatment; or ii) performing a meta-regression where prognostic or diagnostic utility is regressed against the proportion of patients in each study who adhered to treatment. It is recognised however, that meta-regression has low power and is potentially subject to study-level confounding. The second key subgroup/sensitivity analysis of interest will look at the acuteness of cardiovascular disease. This will be achieved by pooling together studies in patients having had an acute coronary syndrome within 12 months, and those presenting with stable coronary artery disease.

### Economic evaluation

#### Systematic review of published cost-effectiveness studies

A systematic review to identify any existing economic studies will be undertaken, including cost analyses, cost-effectiveness, cost-utility and cost-benefit studies, and decision model-based analyses. Relevant outcomes will be quality-of-life, costs and incremental cost-effectiveness ratios. Quality assessment will be appropriate to the study design e.g. the Consensus on Health Economic Criteria (CHEC) list [31] for economic evaluations and the Philips checklist [32] for model-based analyses. If available, economic models will be reviewed and utilised where appropriate to inform a model-based economic evaluation.

#### Health economic modelling

If platelet function testing is demonstrated to have diagnostic/predictive utility (in either the total population or a sub-set), the construction of a cost-effectiveness model will be considered. This will require sufficient evidence of the clinical consequences of testing, compared to no testing, in order to populate the model. Ideally this requires evidence from test-treat trials (specific for a particular test shown to have diagnostic/predictive utility). If such studies exist these will be identified in the systematic reviews above. In the absence of such studies, evidence will have to be obtained from other sources or simulated. The nature of any model will be dependent on the results of the systematic review in terms of the test(s) and population(s) in which diagnostic/predictive utility has been deemed acceptable.

However, in the absence of strong data, a speculative model will be constructed that could be populated with data if future research is conducted. A speculative model will be subject to extensive sensitivity analysis, using a range of hypothetical values in order to demonstrate at which point a test may become cost-effective under a number of test-treat effectiveness assumptions.

The economic evaluation will be carried out from an UK NHS and Personal Social Services (PSS) perspective, to take into account health care costs and longer term social care costs of vascular events. A Markov type, state transition model will be the most appropriate model for this decision problem as it can take into account changes in health states and recurrent events over a long period of time.

The model structure may be informed by existing models which consider antiplatelet therapy for secondary prevention of vascular events. If no such models are identified, models considering other methods of secondary (cardiovascular disease) prevention will be sought.

The model will require data on the prognostic and diagnostic/predictive utility of platelet function tests and subsequent interventions for therapy adjustment, including impact on vascular events, bleeding and mortality. Data will also be required on quality of life and health care costs, in particular the cost of testing, drug costs and acute and long term treatment and care of vascular events and bleeding events. For each important model parameter, a point estimate with a range of possible values (confidence intervals) will be determined and a probability distribution around that point estimate constructed. Base-case evaluation will be based on the most likely estimates. Univariate and probabilistic sensitivity analyses will be conducted to deal with uncertainty in model parameters.

## Discussion

It is anticipated that the results of this project will represent a significant step towards informing clinical practice by indicating whether specific platelet function tests have diagnostic/prognostic utility in a defined population and could thus lead to a reasonably reliable prediction of the risk of clinically important events. This in turn would inform any decisions on whether platelet function testing should be routinely used in specific populations in order to establish a diagnosis of ‘aspirin resistance’ and would further support the investigation of candidate adjustments to therapy to compensate for lack of response to standard aspirin. The strength of ‘aspirin resistance’ based on each test will be determined by its diagnostic/predictive accuracy, its ability to retain prognostic value even after adjustment for other factors, and it retaining good diagnostic/predictive accuracy both in patients who adhered to treatment and those who did not.

If, however, the existing primary studies identified are essentially exploratory in nature, a prospective protocol driven hypothesis-testing study may be required before a change in clinical practice could be justified [33-36]. The results of the proposed review would greatly facilitate the design of such a study. Should this review of primary studies provide little evidence that a specific test in any population has predictive utility, this would point to a requirement for further improvement or refinement in testing methods before platelet testing could be recommended.

We do not seek individual participant data in this review [33,37]. The decision to collate individual patient data (IPD) is premature until a systematic review has established the current reported evidence regarding the individual prognostic and diagnostic accuracy of existing tests, as described above. In the future, where multiple tests are of interest in combination, for example in a prognostic model, then collection of IPD may be more important. However, it is first important to establish the diagnostic/predictive/prognostic ability of individual tests, as, if they have high diagnostic/prognostic accuracy in defined populations, then a prognostic model including multiple variables is potentially not necessary. Thus, this systematic review will establish how suitable individual tests are, and identify whether subsequent research using IPD data is suitable.

## Abbreviations

FN: False negatives; FP: False positives; IPD: Individual patient data; MI: Myocardial infarction; QUADAS-2: Quality Assessment of Diagnostic Accuracy Studies (revised tool); ROC curve: Receiver operating characteristics curve; RCT: Randomised controlled trial; TN: True negatives; TP: True positives

## Competing interests

Marie Lordkipanidze has received speaker honoraria from Eli Lilly who manufacture the antiplatelet agent prasugrel. David Fitzmaurice has received honoraria from Boehringer Ingelheim, Sanofi-Aventis and Astra-Zeneca, but not in relation to antiplatelet therapy. None of the other authors have any competing interests.

## Authors’ contributions

DF is the guarantor. SR, JD and DM drafted the manuscript. All authors contributed to the development of the selection criteria, the risk of bias assessment strategy and data extraction criteria. SB developed the search strategy. RR provided statistical expertise and contributed to the section on analysis. ML provided expertise on platelet function testing. SJ contributed to the section on health economics. All authors read, provided feedback and approved the final manuscript.

## Disclaimer

The views and opinions expressed therein are those of the authors and do not necessarily reflect those of the HTA programme, NIHR, NHS or the Department of Health.
